# Immune Checkpoints as Promising Targets for the Treatment of Idiopathic Pulmonary Fibrosis?

**DOI:** 10.3390/jcm8101547

**Published:** 2019-09-26

**Authors:** JanWillem Duitman, Tom van den Ende, C. Arnold Spek

**Affiliations:** 1 Amsterdam UMC, Center for Experimental and Molecular Medicine, University of Amsterdam, 1105 AZ Amsterdam, The Netherlands; 2 Amsterdam UMC, Department of Medical Oncology, Cancer Center Amsterdam, University of Amsterdam, 1105 AZ Amsterdam, The Netherlands

**Keywords:** immune checkpoints, idiopathic pulmonary fibrosis, PD-L1, IPF

## Abstract

Idiopathic pulmonary fibrosis is a rare, progressive and fatal lung disease which affects approximately 5 million persons worldwide. Although pirfenidone and/or nintedanib treatment improves patients’ wellbeing, the prognosis of IPF remains poor with 5-year mortality rates still ranging from 70 to 80%. The promise of the anti-cancer agent nintedanib in IPF, in combination with the recent notion that IPF shares several pathogenic pathways with cancer, raised hope that immune checkpoint inhibitors, the novel revolutionary anticancer agents, could also be the eagerly awaited ground-breaking and unconventional novel treatment modality limiting IPF-related morbidity/mortality. In the current review, we analyse the available literature on immune checkpoint proteins in IPF to explore whether immune checkpoint inhibition may be as promising in IPF as it is in cancer. We conclude that despite several promising papers showing that inhibiting specific immune checkpoint proteins limits pulmonary fibrosis, overall the data seem to argue against a general role of immune checkpoint inhibition in IPF and suggest that only PD-1/PD-L1 inhibition may be beneficial.

## 1. Introduction

Idiopathic pulmonary fibrosis (IPF) is the most frequent interstitial lung disease with a devastating prognosis for which there is currently no treatment that cures or reverses the disease. IPF is characterized by excessive accumulation of extracellular matrix in the interstitial and alveolar spaces leading to scarring and the destruction of the normal pulmonary epithelium with subsequent breathing difficulties and diminished oxygen uptake [1]. The prevalence of IPF ranges depending on the criteria used for diagnosis but lies somewhere between 3 and 41.8 cases per 100,000 persons [2,3]. Several studies show that the incidence is rising over the last decades, leading to a consequential increase in economic burden on healthcare [2,4,5]. The prognosis of IPF is devastating—median survival after diagnosis is approximately 3 years and its mortality rate thereby exceeds many types of cancer. Treatment modalities for IPF are limited and lung transplantation is the last resort, which is however available for selected patients only. Recently, two novel drugs, that is, pirfenidone and nintedanib, which both slow the decline of lung function in patients with IPF, while pirfenidone also slightly improves survival of IPF patients, became clinically available [6,7,8,9]. Importantly however, both drugs have serious side effects, show no benefit on quality of life and do not stop nor reverse the disease. Novel treatment options are thus eagerly awaited. As future clinical trials are likely to include pirfenidone and/or nintedanib as control treatment, it is envisioned that novel treatment regimens most likely consist of combination therapies in which new drugs are combined with pirfenidone and/or nintedanib [10].

The identification of novel therapeutic targets critically depends on a better understanding of the pathogenesis of IPF and a plethora of studies have consequently been performed to identify pathogenic pathways that may hold promise as targets for therapy. As outlined below, it emerged from these studies that IPF has several pathogenic pathways in common with cancer based on which it has been proposed to treat IPF patients with drugs known to effectively limit cancer progression [11]. The current review explores this interesting and unorthodox view and specifically focuses on the potential relevance of immune checkpoint inhibition, a cancer therapy awarded the 2018 Nobel prize for medicine [12], in the setting of IPF.

## 2. IPF a Cancer-Like Disease

According to the current paradigm, IPF result from an aberrant wound healing response following repetitive epithelial injury. As cancer may be considered a wound that does not heal [13], it has been proposed that IPF should be considered as a neoproliferative disorder of the lung [14]. Both IPF and cancer not only share similar pathways involved in disease progression but also seem to share risks factors. Indeed, smoking, air pollution, occupational exposures and age are shared risk factors between lung cancer and IPF [1,15], whereas uncontrolled proliferation, disturbed cell-to-cell communication, constitutive activation of intracellular signal transduction pathways and resistance to apoptosis are pathogenic mechanisms that are shared between IPF and cancer [16,17,18]. On the contrary, several arguments against IPF as cancer-like disease have been raised of which the lack of somatic mutations and metastasis in IPF may be particularly relevant [19]. Although recent gene set enrichment analysis of IPF and lung cancer databases identified a common pattern of misregulated genes [20], IPF and non-small cell lung cancer (NSCLC) seem largely transcriptionally divergent suggesting that the similarities between IPF and cancer are smaller than envisioned [21].

Despite the ongoing debate whether IPF should be considered a cancer-like disease, several anticancer agents have already successfully been employed in the setting of IPF. The Food and Drug Administration approval of nintedanib, a tyrosine kinase inhibitor used in the management of NSCLC, is obviously the best example of the applicability of cancer drugs in IPF. However, several other drugs developed as anticancer agents, like GSK2126458 (a potent inhibitor of phosphoinositol 3-kinase and mammalian target of rapamycin) and UCN-01 (a competitive ATP inhibitor targeting several kinases that also reactivates FoxO3), show great promise in pre-clinical models of IPF [22,23]. The recent notion that the epithelial cell transforming sequence 2 oncogene, which is highly enriched in NSCLC patients, contributes to epithelial reprogramming in IPF [20], further strengthen the notion that anticancer drugs may hold promise in the treatment of IPF. In the current manuscript, we address this hypothesis by focusing on immune checkpoint inhibition which has revolutionized cancer treatment. Specifically, we explore whether immune check point inhibitors may be the next anticancer drugs successfully employed in IPF.

## 3. Immune Checkpoints in Cancer

Immunotherapy has emerged as a new treatment option for patients with cancer and checkpoint inhibitors are the most commonly used immunotherapy agents [24]. These agents were developed to target so called ‘checkpoints’ located on tumour or immune cells that are commonly used by cancer cells to engage in immune-editing [25]. Evading detection by immune cells is an important hallmark of several cancer types and blocking this process is envisioned to enable immune cells to relocate and engage in cytotoxic killing. Consequently, several agents have been developed to target these checkpoints [25,26]. They either block the programmed death-receptor 1 (PD-1) on T cells (e.g., nivolumab, pembrolizumab) or its ligand programmed-death ligand 1 (PD-L1) on cancer cells (e.g., atezolizumab). Another important checkpoint for which inhibitory agents have been developed is CTLA-4 which is primarily expressed on T cells (e.g., ipilimumab). In the sections below, we will discuss the physiological function of the different checkpoints, describe their role in the treatment of cancer and point to lessons that can be learned for the IPF field. 

### 3.1. PD-1/PD-L1

PD-1 is in normal physiological conditions involved in regulating T cell function [27]. Upon activation of effector T cells (CD8^+^ and type 1 CD4^+^ helper cells) and subsequent secretion of inflammatory cytokines like interferon gamma (IFNy), PD-L1 is upregulated in peripheral tissues [27,28]. Subsequent binding of PD-1 to PD-L1 (or to its second ligand PD-L2) leads to an inhibitory co-stimulatory signal to the T cell receptor (TCR) [29]. This process is vital in preserving immunotolerance and prevents autoimmune pathology. 

In the tumour immune microenvironment (TIME), T cells need to be primed against tumour antigens [30]. Two signals are needed to induce T cell activation: binding of the MHC receptor with the tumour antigen to the TCR and binding of CD80/CD86 (also known as B7-1 or B7-2), which is primarily expressed on antigen presenting cells (APCs), to CD28 expressed by T cells [31]. Upon activation of T cells, immune checkpoints (i.e., PD-1) are upregulated and T cells are consequently instructed to limit cytotoxicity [28]. Depending on tumour type, PD-L1 expression levels on cancer cells are highly variable. Expression of PD-L1 may be cell intrinsically regulated but may also be affected by the secretion of cytokines (IFNy) or by treatment with chemoradiotherapy [27,32,33]. Binding of PD-1 to cancer cell PD-L1 leads to downstream inhibition of the TCR and thereby interferes with the stimulatory signal provided by the MHC presented antigen [29]. This leads to exhausted T cells which are unable to mount an effective immune response. Immune check point inhibitors blocking PD-1 thus prevent the downstream inhibition of TCR signalling and subsequently reinvigorates exhausted CD8^+^ T cells that are again able to engage in cytotoxic killing [25]. Immune check point inhibitors targeting PD-L1 work more or less identical although evidence is emerging that PD-L1 blocking antibodies may also be involved in antibody-dependent cell-mediated cytotoxicity [34]. PD-L1 also shows affinity for the costimulatory CD80 receptor [35]. Blocking PD-L1 prevents the binding of this ligand to CD80 and enables T cells to induce the necessary costimulation upon interacting with antigens. The mechanism of action of PD-L1 blockade may thus not completely overlap with that of PD-1 inhibitors. 

### 3.2. CTLA-4 and Other Checkpoints

CTLA-4 is involved in attenuating T cell activity [36]. Upon binding of the TCR to an MHC presented antigen, CTLA-4 is upregulated [37] and competes with CD28 for binding to the CD80 and CD86 receptors on APCs [36]. When CTLA-4 binds to CD80/CD86, the TCR signal is inhibited [38,39]. Under physiological conditions this process is primarily involved in dampening T cell activity in secondary lymphoid organs and peripheral tissue. CTLA-4 is also expressed on regulatory T cells (Tregs) where it plays a crucial role in immunological tolerance [40,41]. The expression of CTLA-4 on Tregs limits the availability of CD80/CD86 and thus prevents effector T cells from binding to these ligands. 

In cancer CTLA-4 is upregulated when T cells are activated in the TIME or in lymphoid organs [25]. Blocking CTLA-4 restores the positive co-stimulatory signal to T cells mediated through binding of CD28 to the ligands CD80/CD86 expressed by APCs [25]. These APCs can be found in tumour draining lymph nodes or in the TIME [25]. The primary effect of CTLA-4 blockade will be found in the lymph nodes where APCs present their antigens to the T cells. However, tumour-reactive T cells may also benefit from CTLA-4 blockade in the TIME as there may also be APCs present. 

Blockade of CTLA-4 also has distinct effects on the differentiation and content of T cells in the TIME [42]. It seems that CLTLA-4 inhibition leads to the expansion of tumour specific CD8 T cells but also of a subset of exhausted CD8 T cells and Th1 PD1+ICOS+TBET+CD4 effector T cells [42,43]. Moreover, the expression of CTLA-4 on Tregs also leads to the depletion of these cells in the TIME after administration of an anti-CTLA-4 agent serving another mechanism explaining the therapeutic efficacy of CTLA-4 check point inhibitors [44,45].

There are several other checkpoints both on tumour cells, APCs and T cells which can either provide co-stimulatory or co-inhibitory signals to T cells [46]. The following receptors/ligands are expressed on T cells and can improve T cell activation upon binding to its target on tumour cells or APCs: ICOS, 4-1BB, OX40, GITR and CD40L [46]. Co-inhibition can be provided by the following receptors on T cells upon interacting with tumour cells or APCs: TIM-3, BTLA, TIGIT and LAG-3 [46]. For example, TIM-3 is expressed by exhausted T cells but may also interact with NK cell cytotoxicity [47]. This highlights the complex role of these co-signalling pathways and shows they may act upon several different cell types. Ongoing research aims to provide more insight into the role of these alternative checkpoints and to determine whether inhibitors targeting these checkpoints may provide as efficient therapeutic options for patients as the PD-1/PD-L1/CTLA-4 inhibitors.

## 4. What Can We Learn?

Checkpoint inhibitors are now frequently employed across different types of cancer including lung cancer, melanoma and bladder cancer [48,49,50]. In stage IV NSCLC, most relevant for IPF, PD-1/PD-L1 inhibitors are now established treatment options in the first and second line setting [51,52,53]. However, not every patient with NSCLC responds to these agents. The high degree of heterogeneity in the amount of responders can be observed within one tumour type but also between tumour types. Several biomarkers have emerged to predict which patients might benefit from checkpoint inhibitor immunotherapy, although they all have their flaws [30].

The most commonly used marker to predict clinical benefit of checkpoint inhibitor therapy is PD-L1 expression, measured by immunohistochemistry [54]. In several types of cancer, the expression level of PD-L1 on cancer cells may indeed help predict who will benefit. Higher expression levels of PD-L1 (>1% or >50% in lung cancer) are correlated with better response rates [55,56]. However, patients with no detectable PD-L1 expression may still also profit from PD1/PDL-1 inhibitors. 

Patients with a high tumour mutational burden, reflected by the amount of non-synonymous single nucleotide variants or microsatellite instable tumours, also have a higher chance of responding to checkpoint inhibitors [30]. Although not fully understood, checkpoint inhibition is probably more effective in these patients as their tumours present neo antigens which are more immunogenic to T cells [30]. 

Another promising biomarker is the microbiome. Indeed, the diversity and composition of the gut microbiota has been shown to modify checkpoint inhibitor efficacy in pre-clinical models [57,58]. Moreover, increased microbiota diversity was associated with improved immune checkpoint inhibitor response and patients treated with antibiotics during the course of checkpoint inhibitor therapy had decreased antitumor responses [58]. The mechanism of action by which the microbiome affects checkpoint therapy is not fully understood but may be through modulation of cytokine release from the gut thereby influencing the immune response.

Overall, immune checkpoint inhibition truly seems to have revolutionized cancer treatment although its efficacy is highly variable among patients. Unfortunately, there is still no robust biomarker predicting immunotherapy efficacy in cancer and it is envisioned that single biomarkers will never accurately identify patients who will likely benefit from checkpoint therapy [30]. Instead, predictive models that take in account multiple biomarkers are desperately needed for accurate patient selection allowing check point inhibition therapy to live up to its high expectations.

## 5. Immune Checkpoints in IPF

Considering the overlap in pathogenic mechanisms between IPF and cancer and the success of anticancer agents in the treatment of IPF, it is tempting to speculate that immune checkpoint inhibition may also hold promise in the setting of IPF. To prove or refute this hypothesis, we here review available literature on immune checkpoints in IPF. To this end, studies focusing on immune checkpoints in IPF were retrieved from PubMed using the key words listed in Table 1.

### 5.1. PD-1/PD-L1 Axis

**Figure 1:**
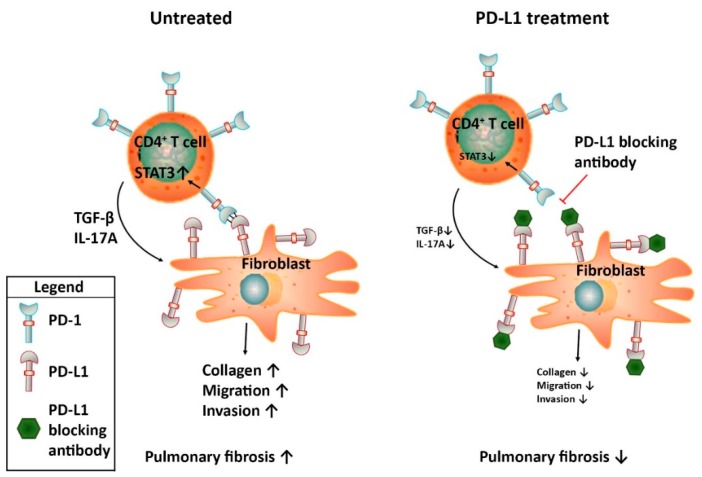
Schematic representation of the proposed mechanism by which the PD-1/PD-L1 axis contributes to pulmonary fibrosis. During IPF, PD-1 is expressed on CD4^+^ T cells whereas PD-L1 is expressed on fibroblasts. The subsequent cross-talk leads to STAT3-mediated IL17A and TGF-β production by the CD4^+^ T cells and pro-fibrotic responses by fibroblasts.

The potential role of the PD-1/PD-L1 checkpoint in IPF has recently been established by showing that the PD-1/PD-L1 axis is induced in IPF patients. In a comprehensive characterization of morphologic and molecular features of pulmonary fibrosis associated cancers, PD-L1 expression was observed in 62% of the patients although the expression analysis in this study was mainly focused on tumour cells [59]. In a pilot study comparing soluble PD-L1 serum levels of IPF patients with healthy controls, a three-fold increase in PD-L1 expression was observed in IPF patients. These results were supported by immunohistochemical analysis of tissue biopsies from IPF lungs in which PD-L1 expression was observed in 9 out of 12 patients [60]. Interestingly, in another study PD-L1 expression in peripheral blood was not increased in IPF patients compared to healthy control but PD-1 expression was increased significantly on T lymphocytes of IPF patients both in peripheral blood and lung tissue [61]. This is in line with the observation that PD-1 surface expression on circulating CD4^+^ T cells and in IPF lung tissue was increased in IPF patients compared to age-matched healthy controls [62]. PD-L1 expression was also detected in lung fibroblasts, with increased levels in the subset of invasive fibroblasts [63]. Irrespective the actual cell type(s) expressing PD-1 or PD-L1, these studies suggest that the PD-1/PD-L1 checkpoint is induced in IPF which is indicative of immune-editing that might affect disease progression. In line with this notion, inhibition of PD-L1 attenuates experimental pulmonary fibrosis in preclinical studies. Bleomycin administration to mice treated with PD-L1 blocking antibodies significantly reduced pulmonary fibrosis [62]. Mechanistic experiments revealed that PD-1 expression on CD4^+^ T cells lead to STAT3 upregulation and subsequent IL-17A and TGF-β expression. Indeed, ex vivo blocking the PD-1/PD-L1 axis resulted in a reduction of STAT3 mediated IL-17A and TGF-β production by CD4^+^ T cells. Interestingly, another study shows that PD-L1 expression on invasive fibroblasts also contributes to pulmonary fibrosis [63]. Genetic ablation or antibody-mediated inhibition of PD-L1 on fibroblasts significantly reduced their invasion and migration in vitro and collagen production in vivo. Although the receptor involved in PD-L1 signalling in vitro remains elusive in the latter study (fibroblasts were negative for PD-1), both studies suggest that the contribution of the PD-1/PD-L1 axis in pulmonary fibrosis is independent of immune regulation and is merely an effect of the cross-talk between CD4^+^ T cells and fibroblasts (see Figure 1). Overall, PD-L1 thus seems to be a promising target to pursue in the quest for new therapeutic options in IPF. Interestingly however, PD-L1 inhibitors should not be used in conjunction with mesenchymal stem cell (MSC) therapy which is currently evaluated as treatment option for IPF in clinical trials [64]. An elegant study actually showed that the beneficial effect of MSCs on pulmonary fibrosis in bleomycin-treated humanized mice is reversed by anti-PD-L1 treatment [61]. Indeed, blocking PD-L1 on MSCs prevents MSC–mediated immunosuppression thereby abolishing the attenuation of pulmonary fibrosis by MSCs. As PD-L1 inhibition most likely does not limit pulmonary fibrosis by its immune modulatory effects, one could however envision to use inhibitors targeting the underlying mediators of PD-L1 signalling relevant in IPF (i.e., STAT3 and/or antibodies directed against IL-17A; see Figure 1) instead of targeting PD-L1 itself. In contrast to the apparent beneficial effect of PD-L1 inhibition in preclinical IPF models, the effect of PD-1 is less evident. Genetic ablation of PD-1 limits bleomycin-induced pulmonary fibrosis [62] but antibody mediated inhibition actually accelerates fibrosis in a humanized pulmonary fibrosis model [65].

### 5.2. CTLA-4 Axis

Another checkpoint inhibitor that has been successfully targeted in NSCLC, especially in combination therapy, is CTLA-4 [66]. Few studies explored the role of CTLA-4 in pulmonary fibrosis but already over a decade ago CTLA-4 was shown to be overexpressed in IPF lungs as compared to Hypersensitivity Pneumonitis lungs [67]. The cellular source of CTLA-4 in IPF lungs remained elusive as CTLA-4 upregulation was deduced from expression profiles obtained from micro-array analysis of total lung homogenates. Kaneko and colleagues [68], however showed that CTLA-4 is expressed by infiltrating lymphocytes in lung tissue of IPF patients. Moreover, they show that the CTLA-4 receptors CD80 (B7-1) and CD86 (B7-2) are expressed on epithelial cells and macrophages in IPF lung biopsies thereby suggesting that the CTLA-4 axis might play a role in the pathogenesis of IPF. In a recent study, anti-CTLA-4 antibody treatment was however shown to aggravate fibrosis in a humanized model of pulmonary fibrosis [65]. It thus seems that CTLA-4 might play a detrimental role in pulmonary fibrosis and that targeting CTLA-4 in IPF patients is not an interesting option to pursue. This claim is however based on a single mouse study and further studies are needed to firmly establish the role of CTLA-4 in pulmonary fibrosis.

### 5.3. TIM-3

The potential role of TIM-3, a novel candidate immune checkpoint protein, on IPF progression was recently studied in the preclinical bleomycin model of pulmonary fibrosis [69]. Anti-TIM-3 antibody treatment aggravated pulmonary fibrosis as evident from increased myofibroblast accumulation, collagen deposition and TGF-β production. TIM-3 was subsequently shown to be expressed on alveolar macrophages where it modified the phagocytic ability resulting in effective clearance of apoptotic cells in lungs. Overall, these results suggest that macrophage TIM-3 limits pulmonary fibrosis and that targeting TIM-3 is not a valid approach in the setting of IPF. Interestingly however, this seems to be at odds with a very recent study suggesting that macrophage TIM-3 contributes to pulmonary fibrosis [70]. Indeed, transgenic overexpression of TIM-3 in macrophages aggravates pulmonary fibrosis by stimulating IL-10 and TGF-β production. Although we do not have a conclusive explanation for the apparent contradictory results, it suggests that high TIM-3 levels obtained by transgenic overexpression differentially affect pulmonary fibrosis as compared to endogenous levels in IPF patients. Despite this latter study, we feel TIM-3 is not an attractive target to pursue in IPF. 

## 6. Immune Checkpoints; a New Horizon or a False Flag?

Based on increased expression levels of immune checkpoint inhibitors in IPF patients, these novel anticancer drugs may seem a promising treatment option to pursue in IPF and indeed several studies have hinted upon this approach. The experimental data however argue against a general role of immune checkpoint inhibition in IPF and suggest that only PD-1/PD-L1 inhibition could be beneficial. The underlying preclinical studies do however not address resolution of fibrosis and/or the treatment in established fibrosis and future clinical studies should therefore elucidate whether PD-1/PD-L1 inhibition also improves patient outcome. Such clinical studies should be carefully designed and controlled as anti-PD-1/PD-L1 therapy increases the risk for severe and potentially life-threatening adverse effects. Specifically, checkpoint inhibitors may induce auto-immune related adverse events like dermatitis, pneumonitis, hypothyroidism and colitis [71,72]. Especially the development of pneumonitis may be burdensome as IPF patients already have (severe) reduced lung function. The incidence of pneumonitis with checkpoint inhibitor monotherapy lies between 3% to 6% in the general cancer population [73]. In line with these numbers, a 2%–5% incidence of pneumonitis was reported in two Checkmate randomized clinical trials (Checkmate 017 and Checkmate 057) comparing nivolumab treatment with docetaxel in NSCLC patients [74,75]. In the KEYNOTE-024 clinical trial, comparing pembrolizumab with platinum-based chemotherapy in NSCLC patients, a comparable incidence of 3-6% of pneumonitis was reported [52]. In the KEYNOTE-010 randomized clinical trial, in which NSCLC patients were treated with pembrolizumab or docetaxel, also a 2%–5% incidence of pneumonitis was observed [76]. Importantly, several studies suggest that the incidence of pneumonitis increases to up to 10% when combining checkpoint inhibitors [73], whereas pre-existing pulmonary fibrosis may also increase the incidence of pneumonitis. Indeed, in a retrospective analysis of 123 NSCLC patients the concomitance of pulmonary fibrosis increased the incidence of pneumonitis from 5.8% to 35.1% during treatment with nivolumab and pembrolizumab [77]. Since there is a 0.2% incidence of death due to pneumonitis in patients treated with checkpoint inhibitors [78], the mortality rate after PD-1/PD-L1 inhibition may increase in IPF patients. More importantly however PD-1/PD-L1 inhibition does not seem to improve pulmonary fibrosis symptoms in NSCLC patients with concomitant IPF. As IPF is an exclusion criterion in clinical studies on immune therapy in lung cancer no conclusive data are available although sketchy case reports of immune check point inhibition in NSCLC patients with concomitant IPF do not show any effect on fibrosis [79,80,81,82,83]. These case reports of NSCLC patients are however not really suited to study the role of PD-1/PD-L1 inhibition in pulmonary fibrosis and future clinical trials should elucidate whether the PD-1/PD-L1 axis holds promise in IPF.

## 7. To Bear in Mind

Although current data do not provide a strong case for immune checkpoint inhibition in IPF at this particular moment, it should be realised that the number of publications focussing on immune checkpoint inhibition in IPF is rather limited and show conflicting results. Studies that aim to clarify the conflicting results, especially with respect to PD-1 and TIM-3, should shed light on the real future of immune checkpoint inhibition in IPF preventing that the baby is thrown out with the bathwater.

In case checkpoint inhibitors will move forward in IPF, it will be important to take into account the heterogeneity of clinical responses observed in cancer patients. Of note, the efficacy of immune therapy is reduced in NSCLC patients with activating mutations in receptor tyrosine kinases [84]. Indeed, in the CheckMate 012, CheckMate153 and KEYNOTE-001 clinical trials a lower response rate was reported in patients with EGFR or anaplastic lymphoma kinase (ALK) positive NSCLC than in those with epidermal growth factor receptor (EGFR) negative and ALK negative NSCLC [84,85,86,87]). As EGFR is upregulated in IPF [88] and may promote fibrotic disease [89], the efficacy of immune checkpoint inhibition may be limited in a subgroup of IPF patients with over-activated tyrosine kinase signalling. In these specific patients, combination therapy of nintedanib and PD-1/PD-L1 inhibitors would be the best option to pursue. 

In addition to mutations in receptor tyrosine kinases, the gut microbiome also determines the clinical efficacy of immune checkpoint inhibition in NSCLC patients. Indeed, based on the diversity and composition of the intestinal microbiota patients can be stratified in responders and non-responders to PD-1 based immunotherapy [90,91]. Although no studies assessed the gut microbiome in IPF, seminal papers show that the lung microbiome is altered in IPF patients and that the bacterial burden affects the pathogenesis and progression of IPF [92,93]. It remains to be determined however whether the lung microbiome may also affect checkpoint inhibition in the setting of IPF.

Overall, it is well conceivable that responses to immune checkpoint inhibition will also highly vary in IPF patients and the identification of biomarkers predicting treatment response is of utmost importance. Although no data are currently available, several patient related characteristics may aid in identifying responders from non-responders. Most obvious, expression levels of checkpoint inhibitors on relevant cell types are likely candidate biomarkers. Alternatively, biomarkers predictive of response in cancer patients may be useful in IPF to select appropriate patients. 

## 8. Conclusions

The success of the anti-cancer agent nintedanib in IPF, in combination with the recent notion of shared pathogenic mechanisms between IPF and cancer, raised enthusiasm that alternative anti-cancer agents could also benefit IPF patients. Based on its revolutionary success in cancer therapy, several investigators addressed the role of immune checkpoints in IPF. The positive results of some landmark studies lead to several advertorials [94,95] claiming that immune checkpoint inhibitors could also revolutionize IPF treatment. However, analysing all available data on immune checkpoint proteins in IPF seems to temper this enthusiasm although targeting PD-L1 may hold promise in IPF. Most likely, PD-L1 inhibition does not act via classical cytotoxic T cell activation in IPF but actually seems to affect the cross-talk between CD4^+^ T cells and fibroblasts resulting in diminished fibrogenesis. 

## Figures and Tables

**Table 1:** Search terms used to select relevant papers focusing on immune checkpoint inhibition in idiopathic pulmonary fibrosis (IPF).

Query in PubMed	# of Publications
PD-1 AND IPF	3
PD-L1 AND IPF	5
PD-1 AND pulmonary fibrosis	13
PD-L1 AND pulmonary fibrosis	11
CTLA-4 AND IPF	1
CTLA4 AND IPF	2
CTLA-4 AND pulmonary fibrosis	11
CTLA4 AND pulmonary fibrosis	7
Immune checkpoint AND IPF	4
Immune checkpoint AND pulmonary fibrosis	13

## References

[1] King T.E., Pardo A., Selman M. (2011). Idiopathic Pulmonary Fibrosis. Lancet.

[2] Strongman H., Kausar I., Maher T.M. (2018). Incidence, Prevalence, and Survival of Patients with Idiopathic Pulmonary Fibrosis in the UK. Adv. Ther..

[3] Hopkins R.B., Burke N., Fell C., Dion G., Kolb M. (2016). Epidemiology and survival of idiopathic pulmonary fibrosis from national data in Canada. Eur. Respir. J..

[4] Navaratnam V., Fleming K.M., West J., Smith C.J., Jenkins R.G., Fogarty A., Hubbard R.B. (2011). The rising incidence of idiopathic pulmonary fibrosis in the UK. Thorax.

[5] Hutchinson J., Fogarty A., Hubbard R., McKeever T. (2015). Global incidence and mortality of idiopathic pulmonary fibrosis: A systematic review. Eur. Respir. J..

[6] Richeldi L., du Bois R.M., Raghu G., Azuma A., Brown K.K., Costabel U., Cottin V., Flaherty K.R., Hansell D.M., Inoue Y. (2014). INPULSIS Trial Investigators. Efficacy and safety of nintedanib in idiopathic pulmonary fibrosis. N. Engl. J. Med..

[7] Crestani B., Huggins J.T., Kaye M., Costabel U., Glaspole I., Ogura T., Song J.W., Stansen W., Quaresma M., Stowasser S. (2019). Long-term safety and tolerability of nintedanib in patients with idiopathic pulmonary fibrosis: Results from the open-label extension study, INPULSIS-ON. Lancet Respir. Med..

[8] King T.E., Bradford W.Z., Castro-Bernardini S., Fagan E.A., Glaspole I., Glassberg M.K., Gorina E., Hopkins P.M., Kardatzke D., Lancaster L. (2014). Ascend Study Group. A Phase 3 Trial of Pirfenidone in Patients with Idiopathic Pulmonary Fibrosis. N. Engl. J. Med..

[9] Noble P.W., Albera C., Bradford W.Z., Costabel U., du Bois R.M., Fagan E.A., Fishman R.S., Glaspole I., Glassberg M.K., Lancaster L. (2016). Pirfenidone for Idiopathic Pulmonary Fibrosis: Analysis of Pooled Data from Three Multinational Phase 3 Trials. Eur. Respir. J..

[10] Wuyts W.A., Antoniou K.M., Borensztajn K., Costabel U., Cottin V., Crestani B., Grutters J.C., Maher T.M., Poletti V., Richeldi L. (2014). Combination Therapy: The Future of Management for Idiopathic Pulmonary Fibrosis?. Lancet Respir. Med..

[11] Vancheri C. (2015). Idiopathic Pulmonary Fibrosis and Cancer: Do They Really Look Similar?. BMC Med..

[12] Hokland P., Hokland M., Cotter F. (2018). The Nobel Prize for Medicine Awarded for Cancer Therapy by Inhibition of Negative Immune Regulation. Br. J. Haematol..

[13] Dvorak H.F. (1986). Tumors: Wounds That Do Not Heal. Similarities between Tumor Stroma Generation and Wound Healing. N. Engl. J. Med..

[14] Vancheri C., Failla M., Crimi N., Raghu G. (2010). Idiopathic Pulmonary Fibrosis: A Disease with Similarities and Links to Cancer Biology. Eur. Respir. J..

[15] De Groot P., Munden R.F. (2012). Lung Cancer Epidemiology, Risk Factors, and Prevention. Radiol. Clin. N. Am..

[16] Ballester B., Milara J., Cortijo J. (2019). Idiopathic Pulmonary Fibrosis and Lung Cancer: Mechanisms and Molecular Targets. Int. J. Mol. Sci..

[17] Tzouvelekis A., Gomatou G., Bouros E., Trigidou R., Tzilas V., Bouros D. (2019). Common Pathogenic Mechanisms between Idiopathic Pulmonary Fibrosis and Lung Cancer. Chest.

[18] Kinoshita T., Goto T. (2019). Molecular Mechanisms of Pulmonary Fibrogenesis and Its Progression to Lung Cancer: A Review. Int. J. Mol. Sci..

[19] Reyfman P.A., Gottardi C.J. (2019). IPF and Lung Cancer: Finding Similarities within Differences. Am. J. Respir. Cell Mol. Biol..

[20] Ulke H.M., Mutze K., Lehmann M., Wagner D.E., Heinzelmann K., Gunther A., Eickelberg O., Konigshoff M. (2019). The Oncogene Ect2 Contributes to a Hyperplastic, Proliferative Lung Epithelial Cell Phenotype in IPF. Am. J. Respir. Cell Mol. Biol..

[21] Spek C.A., Duitman J. (2019). Is Idiopathic Pulmonary Fibrosis a Cancer-Like Disease? Transcriptome Analysis to Fuel the Debate. ERJ Open Res..

[22] Mercer P.F., Woodcock H.V., Eley J.D., Plate M., Sulikowski M.G., Durrenberger P.F., Franklin L., Nanthakumar C.B., Man Y., Genovese F. (2016). Exploration of a Potent PI3 Kinase/Mtor Inhibitor as a Novel Anti-Fibrotic Agent in IPF. Thorax.

[23] Al-Tamari H.M., Dabral S., Schmall A., Sarvari P., Ruppert C., Paik J., DePinho R.A., Grimminger F., Eickelberg O., Guenther A. (2018). Foxo3 an Important Player in Fibrogenesis and Therapeutic Target for Idiopathic Pulmonary Fibrosis. EMBO Mol. Med..

[24] Nishino M., Ramaiya N.H., Hatabu H., Hodi F.S. (2017). Monitoring Immune-Checkpoint Blockade: Response Evaluation and Biomarker Development. Nat. Rev. Clin. Oncol..

[25] Wei S.C., Duffy C.R., Allison J.P. (2018). Fundamental Mechanisms of Immune Checkpoint Blockade Therapy. Cancer Discov..

[26] Boussiotis V.A. (2016). Molecular and Biochemical Aspects of the PD-1 Checkpoint Pathway. N. Engl. J. Med..

[27] Freeman G.J., Long A.J., Iwai Y., Bourque K., Chernova T., Nishimura H., Fitz L.J., Malenkovich N., Okazaki T., Byrne M.C. (2000). Engagement of the PD-1 Immunoinhibitory Receptor by a Novel B7 Family Member Leads to Negative Regulation of Lymphocyte Activation. J. Exp. Med..

[28] Agata Y., Kawasaki A., Nishimura H., Ishida Y., Tsubata T., Yagita H., Honjo T. (1996). Expression of the PD-1 Antigen on the Surface of Stimulated Mouse T and B Lymphocytes. Int. Immunol..

[29] Yokosuka T., Takamatsu M., Kobayashi-Imanishi W., Hashimoto-Tane A., Azuma M., Saito T. (2012). Programmed Cell Death 1 Forms Negative Costimulatory Microclusters That Directly Inhibit T Cell Receptor Signaling by Recruiting Phosphatase Shp2. J. Exp. Med..

[30] Havel J.J., Chowell D., Chan T.A. (2019). The Evolving Landscape of Biomarkers for Checkpoint Inhibitor Immunotherapy. Nat. Rev. Cancer.

[31] Lanier L.L., O’Fallon S., Somoza C., Phillips J.H., Linsley P.S., Okumura K., Ito D., Azuma M. (1995). CD80 (B7) and Cd86 (B70) Provide Similar Costimulatory Signals for T Cell Proliferation, Cytokine Production, and Generation of Ctl. J. Immunol..

[32] Latchman Y., Wood C.R., Chernova T., Chaudhary D., Borde M., Chernova I., Iwai Y., Long A.J., Brown J.A., Nunes R. (2001). Pd-L2 Is a Second Ligand for Pd-1 and Inhibits T Cell Activation. Nat. Immunol..

[33] Chiang S.F., Huang C.Y., Ke T.W., Chen T.W., Lan Y.C., You Y.S., Chen W.T., Chao K.S.C. (2019). Upregulation of Tumor Pd-L1 by Neoadjuvant Chemoradiotherapy (Neocrt) Confers Improved Survival in Patients with Lymph Node Metastasis of Locally Advanced Rectal Cancers. Cancer Immunol. Immunother..

[34] Dahan R., Sega E., Engelhardt J., Selby M., Korman A.J., Ravetch J.V. (2015). FcγRs Modulate the Anti-Tumor Activity of Antibodies Targeting the PD-1/PD-L1 Axis. Cancer Cell.

[35] Butte M.J., Keir M.E., Phamduy T.B., Sharpe A.H., Freeman G.J. (2007). Programmed Death-1 Ligand 1 Interacts Specifically with the B7-1 Costimulatory Molecule to Inhibit T Cell Responses. Immunity.

[36] Walunas T.L., Lenschow D.J., Bakker C.Y., Linsley P.S., Freeman G.J., Green J.M., Thompson C.B., Bluestone J.A. (1994). CTLA-4 Can Function as a Negative Regulator of T Cell Activation. Immunity.

[37] Brunner M.C., Chambers C.A., Chan F.K., Hanke J., Winoto A., Allison J.P. (1999). CTLA-4-Mediated Inhibition of Early Events of T Cell Proliferation. J. Immunol..

[38] Linsley P.S., Greene J.L., Brady W., Bajorath J., Ledbetter J.A., Peach R. (1994). Human B7-1 (CD80) and B7-2 (CD86) Bind with Similar Avidities but Distinct Kinetics to CD28 and CTLA-4 Receptors. Immunity.

[39] Van der Merwe P.A., Bodian D.L., Daenke S., Linsley P., Davis S.J. (1997). CD80 (B7-1) Binds Both CD28 and CTLA-4 with a Low Affinity and Very Fast Kinetics. J. Exp. Med..

[40] Jain N., Nguyen H., Chambers C., Kang J. (2010). Dual Function of Ctla-4 in Regulatory T Cells and Conventional T Cells to Prevent Multiorgan Autoimmunity. Proc. Natl. Acad. Sci. USA.

[41] Wing K., Onishi Y., Prieto-Martin P., Yamaguchi T., Miyara M., Fehervari Z., Nomura T., Sakaguchi S. (2008). CTLA-4 Control over Foxp3+ Regulatory T Cell Function. Science.

[42] Fehlings M., Simoni Y., Penny H.L., Becht E., Loh C.Y., Gubin M.M., Ward J.P., Wong S.C., Schreiber R.D., Newell E.W. (2017). Checkpoint Blockade Immunotherapy Reshapes the High-Dimensional Phenotypic Heterogeneity of Murine Intratumoural Neoantigen-Specific CD8^+^ T Cells. Nat. Commun..

[43] Wei S.C., Levine J.H., Cogdill A.P., Zhao Y., Anang N.A.S., Andrews M.C., Sharma P., Wang J., Wargo J.A., Pe’er D. (2017). Distinct Cellular Mechanisms Underlie Anti-CTLA-4 and Anti-PD-1 Checkpoint Blockade. Cell.

[44] Simpson T.R., Li F., Montalvo-Ortiz W., Sepulveda M.A., Bergerhoff K., Arce F., Roddie C., Henry J.Y., Yagita H., Wolchok J.D. (2013). FC-Dependent Depletion of Tumor-Infiltrating Regulatory T Cells Co-Defines the Efficacy of Anti-CTLA-4 Therapy against Melanoma. J. Exp. Med..

[45] Selby M.J., Engelhardt J.J., Quigley M., Henning J.A., Chen T., Srinivasan M., Korman A.J. (2013). Anti-CTLA-4 Antibodies of IGG2a Isotype Enhance Antitumor Activity through Reduction of Intratumoral Regulatory T Cells. Cancer Immunol. Res..

[46] Fares C.M., van Allen E.M., Drake C.G., Allison J.P., Hu-Lieskovan S. (2019). Mechanisms of Resistance to Immune Checkpoint Blockade: Why Does Checkpoint Inhibitor Immunotherapy Not Work for All Patients?. Am. Soc. Clin. Oncol. Educ. Book.

[47] Ndhlovu L.C., Lopez-Verges S., Barbour J.D., Jones R.B., Jha A.R., Long B.R., Schoeffler E.C., Fujita T., Nixon D.F., Lanier L.L. (2012). TIM-3 Marks Human Natural Killer Cell Maturation and Suppresses Cell-Mediated Cytotoxicity. Blood.

[48] Sul J., Blumenthal G.M., Jiang X., He K., Keegan P., Pazdur R. (2016). FDA Approval Summary: Pembrolizumab for the Treatment of Patients with Metastatic Non-Small Cell Lung Cancer Whose Tumors Express Programmed Death-Ligand 1. Oncologist.

[49] American Association for Cancer Research (2017). Nivolumab Gets FDA Nod for Bladder Cancer. Cancer Discov..

[50] Hazarika M., Chuk M.K., Theoret M.R., Mushti S., He K., Weis S.L., Putman A.H., Helms W.S., Cao X., Li H. (2017). FDA Approval Summary: Nivolumab for Treatment of Unresectable or Metastatic Melanoma Following Progression on Ipilimumab. Clin. Cancer Res..

[51] Pai-Scherf L., Blumenthal G.M., Li H., Subramaniam S., Mishra-Kalyani P.S., He K., Zhao H., Yu J., Paciga M., Goldberg K.B. (2017). FDA Approval Summary: Pembrolizumab for Treatment of Metastatic Non-Small Cell Lung Cancer: First-Line Therapy and Beyond. Oncologist.

[52] Reck M., Rodriguez-Abreu D., Robinson A.G., Hui R., Csoszi T., Fulop A., Gottfried M., Peled N., Tafreshi A., Cuffe S. (2016). Pembrolizumab Versus Chemotherapy for PD-L1-Positive Non-Small-Cell Lung Cancer. N. Engl. J. Med..

[53] Zhou Y., Lin Z., Zhang X., Chen C., Zhao H., Hong S., Zhang L. (2019). First-Line Treatment for Patients with Advanced Non-Small Cell Lung Carcinoma and High Pd-L1 Expression: Pembrolizumab or Pembrolizumab Plus Chemotherapy. J. Immunother. Cancer.

[54] Lu S., Stein J.E., Rimm D.L., Wang D.W., Bell J.M., Johnson D.B., Sosman J.A., Schalper K.A., Anders R.A., Wang H. (2019). Comparison of Biomarker Modalities for Predicting Response to PD-1/PD-L1 Checkpoint Blockade: A Systematic Review and Meta-Analysis. JAMA Oncol..

[55] Ready N., Hellmann M.D., Awad M.M., Otterson A., Gutierrez M., Gainor J.F., Borghaei H., Jolivet J., Horn L., Mates M. (2019). First-Line Nivolumab Plus Ipilimumab in Advanced Non-Small-Cell Lung Cancer (Checkmate 568): Outcomes by Programmed Death Ligand 1 and Tumor Mutational Burden as Biomarkers. J. Clin. Oncol..

[56] Sidaway P. (2019). PD-L1 Positivity Predicts Response. Nat. Rev. Clin. Oncol..

[57] McQuade J.L., Daniel C.R., Helmink B.A., Wargo J.A. (2019). Modulating the Microbiome to Improve Therapeutic Response in Cancer. Lancet Oncol..

[58] Gopalakrishnan V., Spencer C.A., Nezi L., Reuben A., Andrews M.C., Karpinets T.V., Prieto P.A., Vicente D., Hoffman K., Wei S.C. (2018). Gut Microbiome Modulates Response to Anti-PD-1 Immunotherapy in Melanoma Patients. Science.

[59] Guyard A., Danel C., Theou-Anton N., Debray M.P., Gibault L., Mordant P., Castier Y., Crestani B., Zalcman G., Blons H. (2017). Morphologic and Molecular Study of Lung Cancers Associated with Idiopathic Pulmonary Fibrosis and Other Pulmonary Fibroses. Respir. Res..

[60] Jovanovic D., Roksandic M.M., Kotu S.J., Markovic J., Ceriman V., Kontic M., Skodric T.V. (2018). Membrane PD-L1 Expression and Soluble PD-L1 Plasma Levels in Idiopathic Pulmonary Fibrosis-a Pilot Study. J Thorac. Dis..

[61] Ni K., Liu M., Zheng J., Wen L., Chen Q., Xiang Z., Lam K.T., Liu Y., Chan G.C., Lau Y.L. (2018). PD-1/PD-L1 Pathway Mediates the Alleviation of Pulmonary Fibrosis by Human Mesenchymal Stem Cells in Humanized Mice. Am. J. Respir. Cell Mol. Biol..

[62] Celada L.J., Kropski J.A., Herazo-Maya J.D., Luo W., Creecy A., Abad A.T., Chioma O.S., Lee G., Hassell N.E., Shaginurova G.I. (2018). PD-1 up-Regulation on CD4^+^ T Cells Promotes Pulmonary Fibrosis through STAT3-Mediated IL-17a and TGF-beta1 Production. Sci. Transl. Med..

[63] Geng Y., Liu X., Liang J., Habiel D.M., Kulur V., Coelho A.L., Deng N., Xie T., Wang Y., Liu N. (2019). PD-L1 on Invasive Fibroblasts Drives Fibrosis in a Humanized Model of Idiopathic Pulmonary Fibrosis. JCI Insight.

[64] Antoniou K.M., Karagiannis K., Tsitoura E., Tzanakis N. (2017). Mesenchymal Stem Cell Treatment for IPF—Time for Phase 2 Trials?. Lancet Respir. Med..

[65] Habiel D.M., Espindola M.S., Kitson C., Azzara A.V., Coelho A.L., Stripp B., Hogaboam C.M. (2019). Characterization of CD28(Null) T Cells in Idiopathic Pulmonary Fibrosis. Mucosal Immunol..

[66] Pinto J.A., Raez L.E., Oliveres H., Rolfo C.C. (2019). Current Knowledge of Ipilimumab and Its Use in Treating Non-Small Cell Lung Cancer. Expert Opin. Biol. Ther..

[67] Selman M., Pardo A., Barrera L., Estrada A., Watson S.R., Wilson K., Aziz N., Kaminski N., Zlotnik A. (2006). Gene Expression Profiles Distinguish Idiopathic Pulmonary Fibrosis from Hypersensitivity Pneumonitis. Am. J. Respir. Crit. Care Med..

[68] Kaneko Y., Kuwano K., Kunitake R., Kawasaki M., Hagimoto N., Hara N. (2000). B7-1, B7-2 and Class II MHC Molecules in Idiopathic Pulmonary Fibrosis and Bronchiolitis Obliterans-Organizing Pneumonia. Eur. Respir. J..

[69] Isshiki T., Akiba H., Nakayama M., Harada N., Okumura K., Homma S., Miyake S. (2017). Cutting Edge: Anti-TIM-3 Treatment Exacerbates Pulmonary Inflammation and Fibrosis in Mice. J. Immunol..

[70] Wang Y., Kuai Q., Gao F., Wang Y., He M., Zhou H., Han G., Jiang X., Ren S., Yu Q. (2019). Overexpression of TIM-3 in Macrophages Aggravates Pathogenesis of Pulmonary Fibrosis in Mice. Am. J. Respir. Cell Mol. Biol..

[71] Johnson D.B., Chandra S., Sosman J.A. (2018). Immune Checkpoint Inhibitor Toxicity in 2018. JAMA.

[72] Postow M.A., Sidlow R., Hellmann M.D. (2018). Immune-Related Adverse Events Associated with Immune Checkpoint Blockade. N. Engl. J. Med..

[73] Naidoo J., Wang X., Woo K.M., Iyriboz T., Halpenny D., Cunningham J., Chaft J.E., Segal N.H., Callahan M.K., Lesokhin A.M. (2017). Pneumonitis in Patients Treated with Anti-Programmed Death-1/Programmed Death Ligand 1 Therapy. J. Clin. Oncol..

[74] Brahmer J., Reckamp K.L., Baas P., Crino L., Eberhardt W.E., Poddubskaya E., Antonia S., Pluzanski A., Vokes E.E., Holgado E. (2015). Nivolumab Versus Docetaxel in Advanced Squamous-Cell Non-Small-Cell Lung Cancer. N. Engl. J. Med..

[75] Borghaei H., Paz-Ares L., Horn L., Spigel D.R., Steins M., Ready N.E., Chow L.Q., Vokes E.E., Felip E., Holgado E. (2015). Nivolumab Versus Docetaxel in Advanced Nonsquamous Non-Small-Cell Lung Cancer. N. Engl. J. Med..

[76] Herbst R.S., Baas P., Kim D.W., Felip E., Perez-Gracia J.L., Han J.Y., Molina J., Kim J.H., Arvis C.D., Ahn M.J. (2016). Pembrolizumab Versus Docetaxel for Previously Treated, PD-L1-Positive, Advanced Non-Small-Cell Lung Cancer (Keynote-010): A Randomised Controlled Trial. Lancet.

[77] Yamaguchi T., Shimizu J., Hasegawa T., Horio Y., Inaba Y., Yatabe Y., Hida T. (2018). Pre-Existing Pulmonary Fibrosis Is a Risk Factor for Anti-PD-1-Related Pneumonitis in Patients with Non-Small Cell Lung Cancer: A Retrospective Analysis. Lung Cancer.

[78] Haanen J.B.A.G., Carbonnel F., Robert C., Kerr K.M., Peters S., Larkin J., Jordan K. (2017). Esmo Guidelines Committee. Management of Toxicities from Immunotherapy: Esmo Clinical Practice Guidelines for Diagnosis, Treatment and Follow-Up. Ann. Oncol..

[79] Ide M., Tanaka K., Sunami S., Asoh T., Maeyama T., Tsuruta N., Nakanishi Y., Okamoto I. (2018). Durable Response to Nivolumab in a Lung Adenocarcinoma Patient with Idiopathic Pulmonary Fibrosis. Thorac. Cancer.

[80] Kashiwada T., Minegishi Y., Saito Y., Kato T., Atsumi K., Seike M., Kubota K., Terasaki Y., Gemma A. (2019). Organizing Pneumonia after Nivolumab Treatment in a Patient with Pathologically Proven Idiopathic Pulmonary Fibrosis. J. Nippon. Med. Sch..

[81] Khunger M., Velcheti V. (2017). A Case of a Patient with Idiopathic Pulmonary Fibrosis with Lung Squamous Cell Carcinoma Treated with Nivolumab. J. Thorac. Oncol..

[82] Fujimoto D., Morimoto T., Ito J., Sato Y., Ito M., Teraoka S., Otsuka K., Nagata K., Nakagawa A., Tomii K. (2017). A Pilot Trial of Nivolumab Treatment for Advanced Non-Small Cell Lung Cancer Patients with Mild Idiopathic Interstitial Pneumonia. Lung Cancer.

[83] Duchemann B., Didier M., Pailler M.C., Brillet P.Y., Kambouchner M., Uzunhan Y., Freynet O., Chouahnia K., Zelek L., Nunes H. (2019). Can Nivolumab Be Used Safely in Idiopathic Pulmonary Fibrosis?. Rev. Mal. Respir..

[84] Berghoff A.S., Bellosillo B., Caux C., de Langen A., Mazieres J., Normanno N., Preusser M., Provencio M., Rojo F., Wolf J. (2019). Immune checkpoint inhibitor treatment in patients with oncogene-addicted non-small cell lung cancer (NSCLC): Summary of a multidisciplinary round-table discussion. ESMO Open.

[85] Gettinger S., Rizvi N.A., Chow L.Q., Borghaei H., Brahmer J., Ready N., Gerber D.E., Shepherd F.A., Antonia S., Goldman J.W. (2016). Nivolumab Monotherapy for First-Line Treatment of Advanced Non-Small-Cell Lung Cancer. J. Clin. Oncol..

[86] Garon E.B., Hellmann M.D., Rizvi N.A., Carcereny E., Leighl N.B., Ahn M.J., Eder J.P., Balmanoukian A.S., Aggarwal C., Horn L. (2019). Five-Year Overall Survival for Patients with Advanced Non‒Small-Cell Lung Cancer Treated with Pembrolizumab: Results from the Phase I KEYNOTE-001 Study. J. Clin. Oncol..

[87] Leighl N.B., Hellmann M.D., Hui R., Carcereny E., Felip E., Ahn M.J., Eder J.P., Balmanoukian A.S., Aggarwal C., Horn L. (2019). Pembrolizumab in patients with advanced non-small-cell lung cancer (KEYNOTE-001): 3-year results from an open-label.; phase 1 study. Lancet Respir. Med..

[88] Tzouvelekis A., Ntolios P., Karameris A., Vilaras G., Boglou P., Koulelidis A., Archontogeorgis K., Kaltsas K., Zacharis G., Sarikloglou E. (2013). Increased expression of epidermal growth factor receptor (EGF-R) in patients with different forms of lung fibrosis. Biomed. Res. Int..

[89] Epstein Shochet G., Brook E., Eyal O., Edelstein E., Shitrit D. (2019). Epidermal growth factor receptor paracrine upregulation in idiopathic pulmonary fibrosis fibroblasts is blocked by nintedanib. Am. J. Physiol. Cell. Mol. Physiol..

[90] Derosa L., Routy B., Kroemer G., Zitvogel L. (2018). The intestinal microbiota determines the clinical efficacy of immune checkpoint blockers targeting PD-1/PD-L1. Oncoimmunology.

[91] Routy B., Le Chatelier E., Derosa L., Duong C.P.M., Alou M.T., Daillère R., Fluckiger A., Messaoudene M., Rauber C., Roberti M.P. (2018). Gut microbiome influences efficacy of PD-1-based immunotherapy against epithelial tumors. Science.

[92] O’Dwyer D.N., Ashley S.L., Gurczynski S.J., Xia M., Wilke C., Falkowski N.R., Norman K.C., Arnold K.B., Huffnagle G.B., Salisbury M.L. (2019). Lung Microbiota Contribute to Pulmonary Inflammation and Disease Progression in Pulmonary Fibrosis. Am. J. Respir. Crit. Care Med..

[93] Molyneaux P.L., Cox M.J., Willis-Owen S.A., Mallia P., Russell K.E., Russell A.M., Murphy E., Johnston S.L., Schwartz D.A., Wells A.U. (2014). The role of bacteria in the pathogenesis and progression of idiopathic pulmonary fibrosis. Am. J. Respir. Crit. Care Med..

[94] Cedars-Sinai. https://www.cedars-sinai.org/newsroom/study-protein-linked-to-cancer-growth-drives-deadly-lung-disease/.

[95] Pulmonary Fibrosis News. https://pulmonaryfibrosisnews.com/2019/03/25/protein-that-drives-cancer-is-potential-new-therapeutic-target-for-idiopathic-pulmonary-fibrosis-study-suggests/.

